# The Spillover Effects of Extending Liver Transplantation to Patients with Colorectal Liver Metastases: A Discrete Event Simulation Analysis

**DOI:** 10.1177/0272989X241249154

**Published:** 2024-06-03

**Authors:** Hanna Meidell Sjule, Caroline N. Vinter, Svein Dueland, Pål-Dag Line, Emily A. Burger, Gudrun Marie Waaler Bjørnelv

**Affiliations:** Department of Health Management and Health Economics, University of Oslo, Oslo, Norway; Department of Health Management and Health Economics, University of Oslo, Oslo, Norway; Research group for Transplant Oncology, Department of Transplantation Medicine, Oslo University Hospital, Oslo, Norway; Research group for Transplant Oncology, Department of Transplantation Medicine, Oslo University Hospital, Oslo, Norway; Section for Transplantation Surgery, Department of Transplantation Medicine, Oslo University Hospital, Oslo, Norway; Institute of Clinical Medicine, University of Oslo, Oslo, Norway; Department of Health Management and Health Economics, University of Oslo, Oslo, Norway; Center for Health Decision Science, Harvard T.H. Chan School of Public Health; Department of Health Management and Health Economics, University of Oslo, Oslo, Norway; Department of Public Health and Nursing, Norwegian University of Science and Technology, Trondheim, Norway

**Keywords:** liver transplantation, colorectal neoplasms, neoplasm, metastasis, decision modeling, cost and cost analysis

## Abstract

**Background:**

Liver transplantation is an alternative treatment for patients with nonresectable colorectal cancer liver-only metastases (CRLM); however, the potential effects on wait-list time and life expectancy to other patients on the transplant waiting list have not been considered. We explored the potential effects of expanding liver transplantation eligibility to include patients with CRLM on wait-list time and life expectancy in Norway.

**Methods:**

We developed a discrete event simulation model to reflect the Norwegian liver transplantation waiting list process and included 2 groups: 1) patients currently eligible for liver transplantation and 2) CRLM patients. Under 2 alternative CRLM-patient transplant eligibility criteria, we simulated 2 strategies: 1) inclusion of only currently eligible patients (CRLM patients received standard-of-care palliative chemotherapy) and 2) expanding waiting list eligibility to include CRLM patients under 2 eligibility criteria. Model outcomes included median waiting list time, life expectancy, and total life-years.

**Results:**

For every additional CRLM patient listed per year, the overall median wait-list time, initially 52 d, increased by 8% to 11%. Adding 2 additional CRLM patients under the most restrictive eligibility criteria increased the CRLM patients’ average life expectancy by 10.64 y and decreased the average life expectancy for currently eligible patients by 0.05 y. Under these assumptions, there was a net gain of 149.61 life-years over a 10-y programmatic period, which continued to increase under scenarios of adding 10 CRLM patients to the wait-list. Health gains were lower under less restrictive CRLM eligibility criteria. For example, adding 4 additional CRLM patients under the less restrictive eligibility criteria increased the CRLM patients’ average life expectancy by 5.64 y and decreased the average life expectancy for currently eligible patients by 0.12 y. Under these assumptions, there was a net gain of 96.36 life-years over a 10-y programmatic period, which continued to increase up to 7 CRLM patients.

**Conclusions:**

Our model-based analysis enabled the consideration of the potential effects of enlisting Norwegian CRLM patients for liver transplantation on wait-list time and life expectancy. Enlisting CRLM patients is expected to increase the total health effects, which supports the implementation of liver transplantation for CRLM patients in Norway.

**Highlights:**

- Given the Norwegian donor liver availability, adding patients with nonresectable colorectal cancer liver-only metastases (CRLM) to the liver transplantation waiting list had an overall modest, but varying, impact on total waiting list time.
- Survival gains for selected CRLM patients treated with liver transplantation would likely outweigh the losses incurred to patients listed currently.
- To improve the total life-years gained in the population, Norway should consider expanding the treatment options for CRLM patients to include liver transplantation.
- Other countries may also have an opportunity to gain total life-years by extending the waiting list eligibility criteria; however, country-specific analyses are required.

## Introduction

The incidence rate of colorectal cancer in Norway is among highest in the world (e.g., 62 cases per 100,000 individuals in 2022).^1^ Approximately half of patients with colorectal cancer develop metastases, in which the liver is the primary metastatic site and the most frequent cause of death.^2^ Currently, liver resection, in which the liver tumor is surgically removed, has curative potential for patients with colorectal metastases; however, the probability of cancer relapse is high.^3^ For patients with colorectal liver metastases facing a nonresectable disease, palliative chemotherapy is the only treatment option. These patients face a poor prognosis with a 5-y overall survival of approximately 10%.^4,5^

Liver transplantation is considered worldwide to be the standard of care for managing advanced liver diseases. Due to the scarcity of donor organs, not all patients in need of a transplant receive one. Therefore, the tradeoffs associated with expanding the liver transplantation waiting list for new indications of donor eligibility must be carefully evaluated.^6^ Several attempts at liver transplantation for colorectal liver metastatic patients were performed prior to 1995; however, due to poor survival, liver transplantation was abandoned as a treatment option.^7^ During the past 2 decades, survival following liver transplantation in general has improved due to improvements in transplant surgical techniques, imaging, oncologic treatments, and immunosuppressant drugs.^8^ These improvements led a research group at Oslo University Hospital to revisit liver transplantation as an alternative treatment option for patients with nonresectable colorectal liver-only metastases (CRLM). Two nonrandomized pilot trials (i.e., SECA I and SECA II; for details, see Supplementary Appendix 1) examined survival outcomes using alternative liver transplant eligibility criteria for CRLM patients.^2,3^ The results from the SECA I and SECA II trials suggested that liver transplantation for selected CRLM patients yielded promising results, with a 5-y overall survival of 60% and 83%, respectively. This is much higher than the expected survival from currently recommended palliative chemotherapy (10%) for these patients.^4,5^

Recently, Bjørnelv et al.^9^ found that liver transplantation compared with palliative chemotherapy alone for CRLM patients was considered cost-effective for selected patients. As cost-effectiveness is one of the pillars of priority setting in Norway, an intervention shown to be cost-effective is often implemented.^10^ However, due to concerns about liver availability and increasing waiting times for patients currently eligible for liver transplantation, transplantation for CRLM patients in Norway is not currently recommended. As the current liver transplantation waiting times in Norway are low (median waiting time ∼40 d^11^) and the annual number of nonresectable CRLM patients who would be eligible under SECA I (*n* = 4) and SECA II (*n* = 2) criteria are few, the benefits of offering liver transplantation to selected CRLM patients may outweigh the potential harms incurred for patients currently eligible for liver transplantation with conventional transplant indications.

Although the potential health benefits of providing liver transplantation to CRLM patients has been demonstrated,^2,3,9^ information on the impact of expanding the waiting list eligibility on currently listed patients is lacking. Because conducting a randomized control study is neither ethically feasible nor possible given the time required to demonstrate effect, mathematical simulation models provide opportunities to project the expected impacts of expanding wait-list criteria. In the current article, our aim was to develop a model to explore the potential effects on wait-list time and life expectancy of expanding liver transplantation eligibility to include patients with CRLM compared with standard treatment recommendations in Norway.

## Methods

### Analytic Framework

We developed a discrete event simulation (DES) model to reflect the current Norwegian liver transplantation waiting list, including the number of patients on the waiting list, liver availability, and waiting time. The model reflected a patient population consisting of 2 groups: 1) patients currently eligible for the liver transplantation waiting list in Norway (i.e., Status Quo patients) and 2) patients with nonresectable colorectal only-liver metastases (i.e., CRLM patients). We simulated 2 strategies: 1) inclusion of only Status Quo patients on the liver transplant waiting list, while CRLM patients receive standard-of-care palliative chemotherapy, and 2) expanding waiting list eligibility to include CRLM patients. Primary outcomes included median wait-list time, average life expectancy, and the total life-years gained/lost for both patient groups between the different scenarios over a 10-y transplantation program period, tracking the patients included in this 10-y period throughout their remaining lifetime. Secondary outcomes included the number of first transplants, retransplants, exported livers, wait-list withdrawals, and average number of patients in the queue. We calculated the expected effect difference between these strategies by estimating the difference in outcomes between the 2 strategies. We also estimated the break-even point, defined as the point when life-years lost was equal to life-years gained for all patients eligible for liver transplantation under strategy 2. In addition, we estimated the expected marginal effect in waiting times if allowing an increasing number of CRLM patients to be eligible for the liver transplantation waiting list.

### Model Description

**Figure 1:**
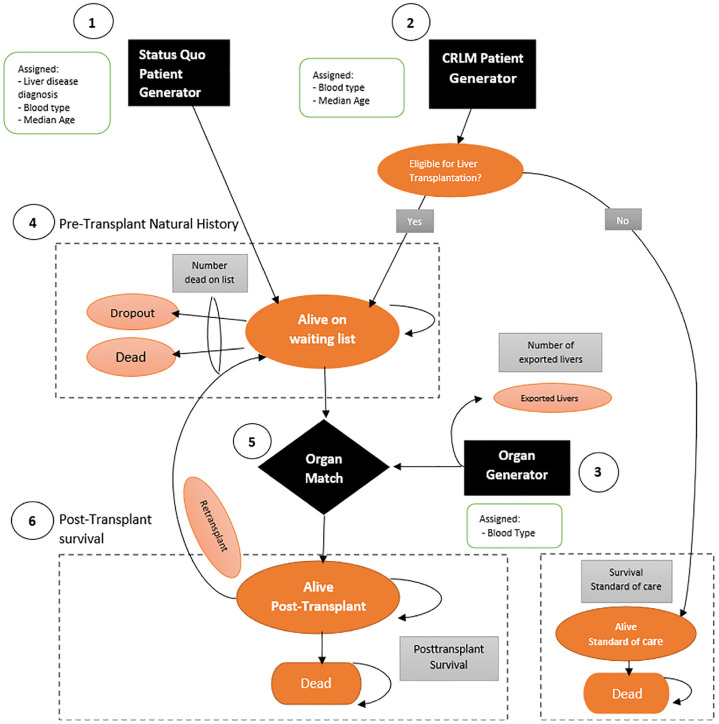
Model schematic of the liver transplantation discrete event simulation (DES) model. The model consists of 6 core modules: 1) Status Quo patient generator module, 2) CRLM patient generator module, 3) pretransplant natural history module, 4) liver generator module, 5) matching module, and 6) posttransplant survival module.

The DES model consisted of 6 core modules: the Status Quo patient generator module, CRLM patient generator module, liver generator module, pretransplant natural history module, matching module, and posttransplant survival module (Figure 1).

#### Status Quo patient generator module

Status Quo patients were generated and placed on the waiting list using a Poisson point process. Consistent with the Poisson point process, the interarrival times between patient listings were exponentially distributed with the estimated daily arrival rate. Directly following generation, patients were assigned a category of liver disease diagnosis. We stratified Status Quo patients by the 5 most frequent liver disease diagnoses in Norway: hepatocellular carcinoma (HCC; 13.7%), primary biliary cirrhosis (PBC; 6.7%), primary sclerosing cholangitis (PSC; 18%), acute liver failure (ALF; 8.3%), and alcoholic cirrhosis (AC; 10.1%).^12^ The remaining patients were pooled together (i.e., “Others”; see Supplementary Table S2 in the Appendix). In addition, we stratified patients according to blood type, which was based on the blood type distribution of the Norwegian population.^13^ Blood type was assigned on the AB0 blood group system. We assumed that the age of the Status Quo patients was equal to the median age associated with the liver disease category, that is, patients diagnosed with PBC, AC, and Others were assumed to be 57 y old; PSC and ALF were assumed to be 43 y old; and HCC patients were assumed to be 61 y old.^11^

#### CRLM patient generator module

CRLM patients were also generated using a Poisson point process, where the interarrival process depended on the number of CRLM patients we simulated (Table 1). Depending on the strategy, CRLM patients were either not eligible for liver transplantation (strategy 1) or eligible for liver transplantation (strategy 2). In strategy 2, CRLM patients were assumed to follow the same liver transplantation pathway as the Status Quo patients. As with Status Quo patients, CRLM patients were assigned a blood type and assumed to have a median age of 57 y.^11,13^

**Table 1:** Model Inputs

Variable	Base-Case Values	Distribution	Source
Interarrival time: Status Quo patients (days)	3.55	Exponential	Melum, 2020^11^
Interarrival time: CRLM patients (days)			
1 CRLM patients annually	365.00	Exponential	Scenario analysis
2 CRLM patients annually^a^	182.50	Exponential	Expert opinion
3 CRLM patients annually	121.67	Exponential	Scenario analysis
4 CRLM patients annually^b^	91.25	Exponential	Expert opinion
5 CRLM patients annually	73.00	Exponential	Scenario analysis
6 CRLM patients annually	60.83	Exponential	Scenario analysis
7 CRLM patients annually	52.14	Exponential	Scenario analysis
8 CRLM patients annually	45.63	Exponential	Scenario analysis
9 CRLM patients annually	40.56	Exponential	Scenario analysis
10 CRLM patients annually	36.50	Exponential	Scenario analysis
Interarrival time between livers (in days)	3.18	Exponential	Melum, 2020^11^
Blood type distribution	A (49%), B (8%), AB (4%), 0 (39%)	Discrete	Heier, 2020^13^
Listed for retransplantation	13.40%	Binomial	Fosby et al., 2015^12^
Receive retransplantation | on being listed	74.90%	Binomial	Fosby et al., 2015^13^
Withdrawn from waiting list per year	6%	Binomial	Expert opinion

CRLM, colorectal cancer liver-only metastases.

a Predicted number of Norwegian CRLM patients eligible under SECA II criteria.

b Predicted number of Norwegian CRLM patients eligible under SECA I criteria.

#### Liver generator module

The generation of livers in the model followed a daily arrival rate based on an average of the annual number of deceased donor livers and number of imported livers to Norway over the past 6 y (Table 1). Directly following generation, livers were assigned blood type based on the Norwegian population with some small distribution adjustments to achieve waiting times consistent with the Norwegian blood type–specific waiting time (for details, see Supplementary Appendix 2).

#### Pretransplant natural history module

Following the generation of Status Quo patients, each patient was assigned time to death and time to dropout on the waiting list, which was dependent on the category of liver disease diagnosis, using the inverse of the cumulative density function (also known as the quantile function; for details, see Supplementary Appendix 2). If a patient’s time of death or dropout occurred before the waiting time needed for transplantation, they were removed from the waiting list. Conversely, if their time to death or dropout exceeded the waiting time, they were eligible for transplantation (refer to the posttransplant natural history module section for details). Time on the waiting list was dependent on the number of patients on the waiting list and the availability of livers. For the strategy in which the CRLM patients were eligible for the waiting list (strategy 2), patients were also assigned a time to death and dropout; that is, the likelihood of being transplanted was determined by the same indicators as for the Status Quo patient group.

#### Matching module

The matching between livers and patients happened with a searching process in the waiting list. Once a donated liver arrived, the searching process began. Based on the AB0 blood group system, the liver was matched with the patient who had been waiting the longest. Following current practice, patients with ALF and patients listed for a re-transplant had a priority in the waiting list and were therefore assigned a liver as soon as a match was found. If a liver was not matched, then the liver was taken out of the model and assumed exported to another Scandinavian country, reflecting the current structure of the Scandinavian liver transplantation program.^14^

#### Posttransplant survival module

The posttransplant survival module determined both posttransplant survival and the need for retransplants in the model. The simulation in the posttransplant survival module began with a probability of the transplantation being unsuccessful, requiring retransplantation. The retransplant listing happened directly following the primary transplantation. However, only patients qualified for a retransplant are relisted on the waiting list and prioritized. Some patient do not qualify for a retransplant, for example, if they have poor general condition, severe comorbidity, uncontrollable infection, or other malignancy. These were removed from the model and their life-years were estimated at the time of removal. Patients who received a successful primary transplantation or a successful retransplantation were randomly assigned a time to death according to the posttransplant disease-specific diagnosis survival distribution, using the quantile function. The patients who were pooled together in the group “Others” were assigned a time to death according to the general overall posttransplant survival. If the patient died within 10 y posttransplant, the patient was removed from the model and their life-years were recorded. If the patient was alive >10 y posttransplantation, the time to death was updated to be equal to the background age-specific mortality (i.e., we assumed no excess liver transplant associated mortality after 10 y, which is consistent with available survival curves).^11,12^ Background mortality was assigned to patients depending on their age. For CRLM patients, if eligible for the waiting list, the same approach as described above applied. For more details on model assumptions, see Supplementary Appendix 4.

### Data Inputs

To reflect the current waiting list in Norway, the interarrival times of currently listed patients and liver availability were based on the number of listed patients and liver availability from Scandiatransplant (Table 1).^14^ The daily arrival rate was informed by the average number of patients enlisted and the average number of available livers during the last 6 y in Norway. Input data related to retransplantation were informed by Fosby et al.,^12^ while posttransplant survival was estimated using Fosby et al.^12^ and the annual report from Nordic Liver Transplant Registry^11^ using the most updated available survival estimates for each liver disease diagnosis. Posttransplant survival for CRLM patients was informed from the SECA I and SECA II trials.^3,15^ The survival estimates for CRLM patients receiving palliative chemotherapy was based on the Nordic Vll trial,^16^ using the same selected cohort of patients from the trial as Dueland et al.^17^ Inputs used for risk of death and dropout on waiting lists were based on cumulative incidence curves from American studies on different liver disease diagnoses.^17–21^ Background mortality was informed by Norwegian national life tables.^22^

Data used for the survival analyses were extracted from published Kaplan-Meier curves and cumulative incidence curves using WebPlotDigitizer (for details, see Supplementary Appendix 5). Data for 9 survival analyses were extracted from published Kaplan-Meier curves related to survival after treatment (posttransplantation or palliative chemotherapy). In addition, data for 8 survival analyses were extracted from published cumulative incidence curves related to risk of death and dropout. Lastly, 3 survival analyses related to background mortality (at ages 43, 57, and 61 y) were conducted. For all survival analyses, we assessed the goodness of fit from 5 parametric distributions: the exponential, Weibull, log-logistic, log-normal, and Gompertz following NICE guidelines,^23^ using visual inspection, shape of the hazard function, the Akaike information criterion, and the Bayesian information criterion. For details, see Supplementary Appendix 6.

### Analyses

#### Model validation

Consistent with validation definitions in Eddy et al.,^24^ we assessed the model’s face validity through collaboration with Norwegian clinicians with expertise on liver transplantation (authors P.D.L. and S.D., principal investigators of SECA I and II). We assessed internal validity by testing that the model’s inputs and outputs matched expectations and data used in the development of the model. We also compared the model outcomes with published outcomes (i.e., median waiting times from our model were compared with the median waiting time by blood type in Norway). We compared our model survival inputs and outputs with the extracted published Kaplan-Meier curves, and other waiting list statistics were compared with the published data used (see Supplementary Appendix 8).

#### Base-case analysis

Our base-case analysis evaluated 2 strategies, strategy 1 and strategy 2, in which liver transplantations over a 10-y programmatic period were simulated. After running preliminary analyses to identify the burn-in period to stabilize the wait time in the queues and the number of replications required to reduce stochastic uncertainty, analysis outcomes were counted after a 1,900-d burn-in period and were averaged over 7,000 replications (see Supplementary Appendix 7). While the analysis captured 10 y of transplants under each strategy, we account for the health benefits accrued for the patients simulated within the 10-y period over their remaining lifetime. The analyses were stratified by SECA I and SECA II eligibility criteria, and analyses are presented separately. The primary model outcomes included life-years for Status Quo and CRLM patients (average life expectancy and total life-years over the 10-y programmatic period), in addition to median waiting time by blood type. Median waiting time was reported from the model, consistent with output available from empirical data. Analyses reflected the expected annual number of CRLM patients who qualify in Norway under SECA I (*n* = 4) or SECA II (*n* = 2) eligiblity criteria. In addition, we identified the maximum acceptable number of CRLM patients who could be eligible for the waiting list, defined as the break-even point at which the expected total life-years gained for CRLM patients was equal to the expected life-years loss for Status Quo patients (over a 10-y programmatic period). Guided by expert opinion, we explored up to a maximum of 10 CRLM patients per year, as it is unrealistic that >10 CRLM patients per year would qualify under SECA I or SECA II. In addition to total life-years and median waiting time, we also evaluated the impact of expanding waiting list eligibility on additional secondary outcomes, including the annual number of first transplants, retransplants, exported livers, wait-list withdrawals, and average number of patients in the queue. These results were not dependent on SECA criteria; therefore, we have not distinguished these results by SECA criteria. After running preliminary analyses to identify the burn-in period to stabilize the wait time in the queues and the number of replications required to reduce stochastic uncertainty, analysis outcomes were counted after a 1,900-d burn-in period and were averaged over 7,000 replications (see Supplementary Appendix 7).

#### Uncertainty analyses

We conducted one subgroup analysis and several uncertainty analyses. The subgroup analysis was conducted on selected patients within the SECA I trial: the selection of these patients lies between SECA I and SECA II criteria. Also, to analyze how survival assumptions affected our findings, we conducted structural uncertainty analyses with different parametric survival specifications for SECA I, SECA II, and palliative chemotherapy. For these analyses, we changed the base-case assumption of a log-logistic parametric distribution to assume a Weibull distribution, which showed reasonable fit to the data yet poorer survival. We additionally conducted 2 uncertainty analyses to evaluate how liver availability affected the waiting time for liver transplantation and other outcomes. The base-case liver availability used in the model was compared with a lower (103 livers) and higher (121 livers) liver availability, which we refer to as the worst- and best-case uncertainty analysis, respectively.

The model was developed in Arena Simulation Software (version 16.10). Stata SE 16.1 was used to analyze the individual-level data reported from Arena Simulation Software and to conduct survival analyses. Arena code may be made available upon reasonable request to Emily Burger, emily.burger@medisin.uio.no.

## Results

### Model Validation

Compared with empirical estimates, our model outputs were consistent with most validation outcomes (Table 2; Supplementary Appendix 8). Importantly, the median waiting time by blood type was nearly identical, with all waiting times within the range of median wait time in the past 5 y.^14^

**Table 2:** Internal Validation in Terms of the Model Outcomes and Median Waiting Times in the Model Compared with the Average Numbers Reported in Norway (Rounded to the Nearest Whole Number)

Variable	Model Average (Average Range^a^) (per Year)	Average (Range^b^) in Norway in the Past 6 y (per Year)
Number enlisted for first transplantation	103 (92–115)^a^	103 (81–119)
First liver transplantations	80.8 (72–91)^a^	81 (72–88)
Retransplantations	9.0 (6–13)^a^	12 (8–19)
Withdrawals (independent of waiting time)	6.2 (3–9)^a^	7 (5–13)
Death/dropout Status Quo	3.3 (1–8)^a^	5 (3–7)
Total withdrawals from waiting list	9.5	12
Deceased donor livers	95	95 (83–102)
Imported livers	20	19 (12–27)
Deceased donor livers + imported livers	115 (100–127)^a^	114
Exported livers	11 (8–27)^a^	22 (20–28)
Average number waiting	19.5 (0–106)^c^	22 (15–34)
Waiting Time by Blood Type	Median (Range^c^) in Model (d)	Median (Range^a^) in Norway in Past 5 y (d)
A	32 (0–390)	32 (26–42)
B	53 (0–357)	48 (40–63)
AB	21 (0–356)	21 (14–26)
0	106 (0–794)	92 (66–111)
Overall	52 (0–794)	40 (26–62)
Survival after Transplantation	75% Overall Survival in Model (y)	75% Overall Survival Empirical (y)
HCC	2.20	2.32
PBC	7.25	8.75
PSC	8.94	8.71
ALF	2.23	2.42
AC	5.43	6.48
Others	5.05	5.50
Palliative chemotherapy	1.18	1.29
CRLM SECA I	2.64	2.83
CRLM SECA II	5.06	N/A

AC, alcoholic cirrhosis; ALF, acute liver failure; CRLM, colorectal cancer liver-only metastases; HCC, hepatocellular carcinoma; PBC, primary biliary cirrhosis; PSC, primary sclerosing cholangitis.

a Minimum average and maximum average across 7,000 replications.

b Minimum and maximum over the past 6 y in Norway.

c Minimum and maximum across all 7,000 replications.

### Base-Case Analyses

#### Median waiting time

**Figure 2:**
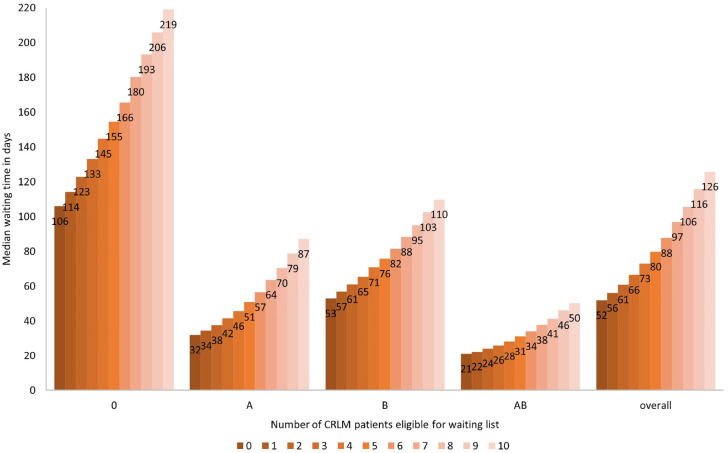
Change in median waiting time by blood type when allowing colorectal cancer liver-only metastases patients to be eligible for the liver transplantation waiting list.

The model projected an increasing median waiting time as additional CRLM patients were eligible for the liver transplantation waiting list. For example, when we simulated the expected annual number of CRLM patients under SECA II (average 2 per year) or SECA I (average 4 per year), the overall median waiting times increased from 52 d to 61 d and from 52 d to 73 d, respectively, compared with strategy 1 (no liver transplantations for CRLM patients). There were variations in the number of additional days patients had to wait by blood type. For example, the wait-list time for patients with blood type 0 increased from 106 to 123 d (an additional 17 d) and from 106 to 145 d (an additional 39 d) when, respectively, 2 CRLM and 4 CRLM patients were eligible for the waiting list. In contrast, the wait-list time for patients with blood type AB increased from 21 to 24 d (an additional 3 d) and from 21 to 28 d (an additional 7 d) (Figure 2) under the same conditions.

#### Health benefits under SECA I eligibility criteria

**Figure 3:**
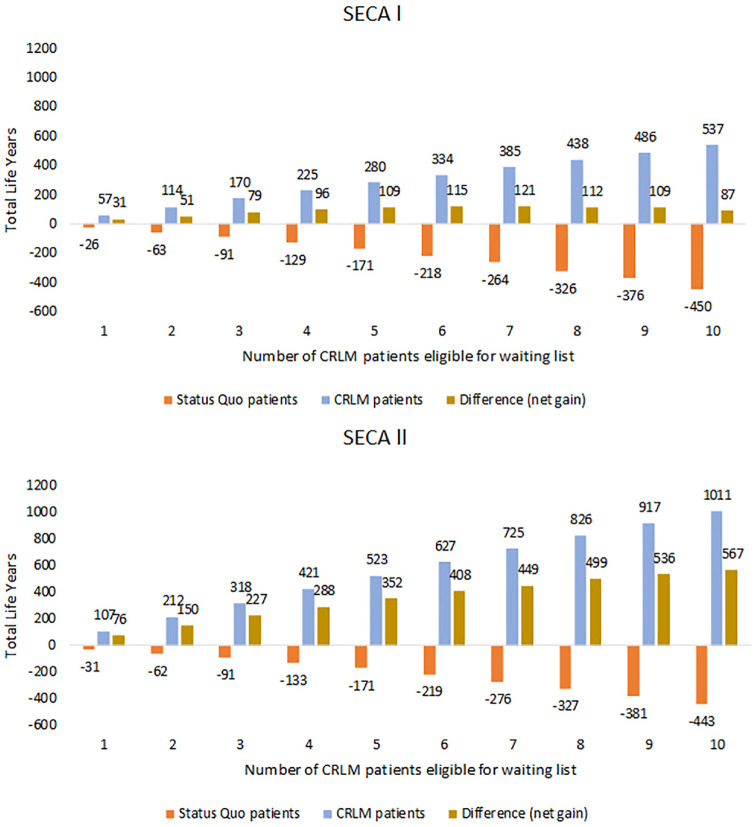
Total life-years lost/gained over a 10-y transplantation period as the number of selected colorectal cancer liver-only metastases (CRLM) patients eligible for transplantation increased. The brown bar represents the aggregated life-years for a patient currently eligible (Status Quo patients) and a CRLM patient, that is, potential spillover effects by allowing CRLM patients reflecting SECA I (A) and SECA II (B) eligibility criteria to be enlisted on the waiting list. The orange bar represents the expected life-years loss for Status Quo patients (*n* = 103 annually). The blue bar represents the expected life-years gained for the CRLM patients.

Over a 10-y programmatic transplantation period for strategy 1 (when CRLM patients were not eligible for liver transplantation), we found that the remaining average life expectancy was 15.28 y for a Status Quo patient and 2.48 y for a CRLM patient (Table 3). If the CRLM patient eligibility criteria for the liver transplantation waiting list reflected the criteria from the SECA I trial, adding an average of 4 CRLM patients on the list increased their average life expectancy from 2.48 to 8.12 y. However, we found that as the number of CRLM patients placed on the waiting list increased, the Status Quo patients continued to lose life-years due to increasing waiting times (Figure 3a, orange bar). For example, under the assumptions that 4 CRLM patients were added to the waiting list each year, we projected that each Status Quo patient would lose an average of 1 mo (0.12 life-years). When we aggregated the total remaining life-years for both patient groups (assuming 4 CRLM transplants per year) over a 10-y programmatic period, we found that extending transplantation to CRLM patients yielded positive net gains (an additional 96 life-years) over all patients. Total life-years peaked when including 7 CRLM patients (121 life-years) (Figure 3a, brown bar) and continued to outweigh the life-years lost for Status Quo patients.

**Table 3:** Average Remaining Life Expectancy per Patient by Transplantation Strategy

	No. of CRLM Patients	Status Quo Patients	Palliative Chemotherapy	Palliative Chemotherapy
Strategy 1	0	15.28	2.48	2.48
		Status Quo Patients	CRLM Patients (SECA I)	CRLM Patients (SECA II)
Strategy 2	1	15.25	8.19	13.16
	2	15.23	8.17	13.12
	3	15.20	8.15	13.12
	4	15.16	8.12	13.07
	5	15.13	8.11	13.01
	6	15.09	8.06	12.99
	7	15.06	8.03	12.93
	8	15.01	7.91	12.86
	9	14.97	7.93	12.76
	10	14.93	7.92	12.71

CRLM, colorectal cancer liver-only metastases.

#### Health benefits under SECA II eligibility criteria

When we further restricted the CRLM patients’ eligibility criteria consistent to SECA II, we found that although the level of expected life-years lost for Status Quo patients remained the same as under the SECA I criteria, the expected life-years gained per transplanted CRLM patient was higher for SECA II compared with SECA I (Figure 3b, brown bar). For example, when adding an average of 2 CRLM patients on the list (the expected annual number of Norwegian CRLM patients eligible under the SECA II criteria), the model projected each CRLM patient to have an average life expectancy of 13.12 y, increasing these patients’ life expectancy by 10.64 y compared with strategy 1 (Table 3). When we aggregated life-years for both patient groups, the expected life-years gained by including CRLM patients on the transplantation list never outweighed the expected life-years lost for the Status Quo patients from increasing their transplant wait time (Figure 3b, brown bar). For more details, see Supplementary Appendix 9.

#### Secondary outcomes

As expected, the average number of patients in queue on the waiting list and the number of annual withdrawals increased as the number of annual eligible CRLM patients increased. Consequently, the annual number of exported livers decreased while the annual number of first transplants increased. For example, if 2 CRLM patients were listed, we found that the average number in queue increased from 19.5 to 22.7 patients and the average annual number of first transplants increased from 80.8 to 82.1, whereas the number of exported livers reduced from 11.1 to 9.4 (Table 4). These secondary outcomes remained the same irrespective of applying the SECA I or SECA II CRLM transplantation eligibility criteria.

**Table 4:** Model Outcomes (Yearly Average over a 10-y Programmatic Period)

Model Outcome	No. of CRLM Patients Eligible										
	0	1	2	3	4	5	6	7	8	9	10
First transplants	80.8	81.5	82.1	82.8	83.4	84.9	84.5	85.0	85.6	86.0	86.4
Retransplants	9.0	9.1	9.2	9.2	9.3	9.4	9.4	9.5	9.5	9.6	9.6
Withdrawals from waiting list	6.2	6.2	6.2	6.2	6.2	6.2	6.2	6.2	6.2	6.2	6.2
Death/dropout Status Quo	3.3	3.4	3.6	3.8	4.0	4.2	4.4	4.7	5.0	5.2	5.5
Total withdrawals from waiting list	9.5	9.6	9.9	10.0	10.2	10.4	10.6	10.9	11.2	11.4	11.7
Death/dropout CRLM	0.0	0.0	0.0	0.1	0.1	0.1	0.2	0.3	0.3	0.4	0.5
Exported livers	11.1	10.2	9.4	8.5	7.7	7.0	6.3	5.6	5.0	4.4	3.9
Average number waiting (based on 10 y)	19.5	21.0	22.7	24.6	26.8	28.9	31.3	34.1	36.8	39.7	42.7

CRLM, colorectal cancer liver-only metastases.

### Uncertainty Analyses

In the subgroup analysis when we explored eligibility criteria between SECA I and II, we found that when including the expected annual number of 4 selected CRLM patients, we estimated the aggregated life to increase from 96 life-years in our base case to 137 life-years over a 10-y programmatic period (see Supplementary Appendix 10). In our structural uncertainty analysis in which we varied the posttransplantation parametric survival specifications to yield poorer survival for CRLM patients, we found that aggregated life-years decreased. In contrast, when we assumed that palliative chemotherapy yielded poorer survival, we found that aggregated life-years increased (Table 5).

**Table 5:** Uncertainty Analyses^a^

Net Life-Years	No. of CRLM Patients (under SECA I)					No. of CRLM Patients (under SECA II)				
Scenario	2	4	6	8	10	2	4	6	8	10
Base case (10-y time horizon)	51	96	115	112	87	150	288	408	499	567
SECA I and SECA II assuming Weibull	37	54	64	36	7	153	277	400	487	552
Palliative chemotherapy assuming Weibull	55	105	128	130	109	154	296	421	516	589
Better overall survival for Status Quo patients	47	62	71	87	35	118	239	387	448	510
Base case (7-y time horizon^b^)	36	66	76	74	61	103	201	286	342	388
Best-case scenario	76	149	209	254	298	178	351	518	659	786
Worst-case scenario	−69	−156	−334	−653	—	31	32	−73	−256	—

—, not able to conduct the analysis, see Supplementary Appendix 11; CRLM, colorectal cancer liver-only metastases.

a In the structural uncertainty analysis labeled “Better overall survival,” all of the Status Quo patients were assigned the survival based on the diagnosis with the overall best survival (primary sclerosing cholangitis). In the scenario analysis labeled “SECA I and SECA II assuming Weibull,” the posttransplant survival for the Status Quo patients was based on the Weibull distribution, both for the SECA I and the SECA II analyses. In the scenario analysis labeled “Palliative chemotherapy assuming Weibull,” the survival for the CRLM patients when receiving palliative chemotherapy was based on the Weibull distribution. The scenario labeled “Best-case scenario” assumed on average 121 livers available annually, and the “Worst-case scenario” assumed 103 liver available annually.

b For an explanation of 7-y time horizon, see Supplementary Appendix 11.

The results of the scenario analysis, which examined the best case and the worst case in terms of liver availability, were different according to the SECA eligibility criteria. For SECA I and SECA II, in the best-case scenario, which assumed 121 livers were available annually in Norway instead of 115 livers we found an increasing aggregated life expectancy in line with an increasing number of CRLM patients eligible for the liver transplantation waiting list. Under this best-case scenario, a peak in aggregated life-years for the 2 patient groups was never achieved compared with the base-case analysis, in which the peak was reached after including 7 CRLM patients on the waiting list annually. In the worst-case scenario (103 livers available annually, on average), for SECA I, the aggregated expected life-years under the SECA I eligibility criteria were projected to decrease immediately; in contrast, if using the eligibility criteria from the SECA II trial, the results were projected to yield net positive gains up to 4 CRLM patients before an immediate decrease (Table 5). When we estimated the median waiting time under the best-case and worst-case scenario assumptions, we found that the median waiting time increased when the annual liver availability decreased (see Supplementary Appendix 11).

## Discussion

Our study has demonstrated how a DES simulation model can be developed to explore the impact on wait-list time and life expectancy on patients currently eligible for liver transplantation by extending liver transplantation to selected CRLM patients in Norway. Our results showed that adding CRLM patients to the waiting list increased the overall median waiting time for patients currently on the waiting list; however, the expected life-years gained for CRLM patients offered liver transplantation outweighed the expected life-years lost due to increased wait-list time for Status Quo patients. Our findings were most robust when CRLM patients were selected for liver transplantation using the more restrictive SECA II eligibility criteria. Consequently, when we aggregated the life-years for CRLM and Status Quo patients under realistic scenarios of the number of enlisted CRLM patients, expansion of the eligibility criteria improved the overall health in the population (i.e., the net life-years were positive compared with maintaining current recommendations).

Through our simulations, we identified a peak point of 7 CRLM patients, when assuming eligible CRLM patients based on patient characteristics and survival reflecting SECA I criteria. In contrast, when we used patient characteristics and survival reflecting the SECA II trial, the model projected that no break-even point existed up to 10 new patients per year, far exceeding the number of expected patients in Norway. The differences between SECA I and SECA II can be explained by the poorer survival in SECA I compared with SECA II. However, in the subgroup analysis, when we assumed a more selective eligibility criteria for the subgroup within the SECA I trial, we found that offering transplantation to CRLM patients could almost double the life-years gained compared with no selection within SECA I. This subgroup analysis highlights that the inclusion criteria in SECA II may be too restrictive if the objective is to maximize total life-years.

Several simulation models have been developed to address liver transplantations.^25–27^; however, to our knowledge, only 1 has been developed to assess the expansions of transplantation eligibility to include HCC patients.^28^ They found that an expansion of the eligibility criteria would require a survival rate of approximately 61% at 5 y after transplantation for the newly eligible patients. Although not directly comparable with our study, these analyses demonstrate alternative methods of how decision making related to allocation of scarce donors may be evaluated.

Our analyses have several limitations worth mentioning. Norwegian guidelines recommend quality-adjusted life-years (QALYs) as outcome measures for effectiveness when assessing health care interventions.^29^ Our current analysis did not consider QALYs due to lack of available data on patients’ health-related quality of life; however, including both QALYs and economic costs will be the focus of future model expansions.

Our model relied on several simplifying assumptions. First, the matching between patient and livers could have been based on additional matching criteria than the blood type alone. In reality, the matching between livers and patients is more complex, in which the clinicians decide with whom the liver should be matched with using factors such as patient body size, severity of disease, and the quality of the liver^30^; thus, the clinicians look at the entirety of the waiting list and do not just follow the queuing system “first in, first out” based on blood type (although with prioritization of patients with acute liver failure or listed for retransplants). Consequently, a match between liver and patient will occur more easily in the model, which has been shown in the results in terms of fewer exported livers and increased number of first transplanted patients. However, including these extended matching criteria would increase the complexity of the model drastically, and we do not believe that this would affect the outcome as we have managed to achieve a similar waiting time as the empirical data. Importantly, model complexity should be weighed against model transparency; we believe more matching criteria would reduce transparency without increasing the precision of the model. Future expansions of the current model could incorporate patient-leveled data from SECA I, SECA II, and the Scandiatransplant-registry.^3,14,15^ Imported and exported livers could be interdependent, as there are approximately the same number each year.^31^ As the number of imported livers is modeled together with the number of deceased donor livers, the number of livers will not be reduced even though exported livers are reduced in the model. Lastly, although live liver donation may be an option to reduce queuing burden, in Norway, living donations are restricted to children only^32^ and therefore not relevant in the short term for Norwegian policy.

The assumption that patients die as soon as they are removed from the waiting list may underestimate the total absolute life-years under all scenarios of transplantation. However, the proportion who are removed from the waiting list is small (approximately 6% per year), while the median wait-list time is on average 52 d. Therefore, we expect the underestimation of total life-years to be small. In addition, as both the Status Quo and CRML patients are subject to the same assumption, the expected incremental differences between the current eligibility criteria (no CRLM) and increasing the number of CRLM patients under different scenarios is even smaller and unlikely to affect our conclusions; if they do, they will be slightly in disfavor of enlisting CRLM patients.

Despite these limitations, we took several steps to ensure our model produced outputs consistent with real-world data. First, the internal validation (Table 2) confirmed that our model has been able to recreate a similar structure as the current Norwegian liver transplantation waiting list. Second, the data used for the CRLM patients in the model were based on data from the newest trials available. Third, no study has previously analyzed the effects on wait-list time and life expectancy of allowing selected CRLM patients to be eligible for the liver transplantation waiting list. Subsequently, our DES model is novel and of scientific relevance, not only in Norway but also in other countries evaluating similar policies.

In conclusion, we found that given the Norwegian donor liver availability, adding CRLM patients to the liver transplantation waiting list had an overall modest, but varying, effect on waiting list time. After including these effects, we found that the survival gains for selected CRLM patients treated with liver transplantation would likely outweigh the losses incurred to patients listed currently. To improve the total life-years gained in the population, Norway should consider expanding the treatment options for CRLM patients to include liver transplantation. Other countries, despite their higher wait-list death and waiting time, may also have an opportunity to gain total life-years by extending the waiting list eligibility criteria; however, country-specific analyses are required.

## Supplemental Material

sj-docx-1-mdm-10.1177_0272989X241249154 – Supplemental material for The Spillover Effects of Extending Liver Transplantation to Patients with Colorectal Liver Metastases: A Discrete Event Simulation Analysis Supplemental material, sj-docx-1-mdm-10.1177_0272989X241249154 for The Spillover Effects of Extending Liver Transplantation to Patients with Colorectal Liver Metastases: A Discrete Event Simulation Analysis by Hanna Meidell Sjule, Caroline N. Vinter, Svein Dueland, Pål-Dag Line, Emily A. Burger and Gudrun Marie Waaler Bjørnelv in Medical Decision Making
