# Supplementary material for: The Spillover Effects of Extending Liver Transplantation to Patients with Colorectal Liver Metastases: A Discrete Event Simulation Analysis
Source: Med Decis Making. 2024 Jun 3;44(5):529–42. doi: 10.1177/0272989X241249154 (PMC11283734; doi:10.1177/0272989X241249154)
Supplement: sj-docx-1-mdm-10.1177_0272989X241249154 – Supplemental material for The Spillover Effects of Extending Liver Transplantation to Patients with Colorectal Liver Metastases: A Discrete Event Simulation Analysis [file sj-docx-1-mdm-10.1177_0272989X241249154.docx]

Supplementary Appendix to accompany:

**Impact of extending liver transplantation to patients with colorectal liver metastases on waitlist time and life expectancy: *A discrete event simulation analysis***

**Table of contents**

[Supplementary Appendix 1 – information on SECA studies 3](#_Toc153969123)

[Supplementary Appendix 2 - Liver blood type distribution 4](#_Toc153969124)

[Supplementary Appendix 3– Quantile functions 5](#_Toc153969125)

[Supplementary Appendix 4 - Model assumptions 6](#_Toc153969126)

[Supplementary Appendix 5 - Reconstructing time-to-event data 9](#_Toc153969127)

[Supplementary Appendix 6 - Survival analyses 10](#_Toc153969128)

[Supplementary Appendix 7 - Stochastic uncertainty 14](#_Toc153969129)

[Supplementary Appendix 8 - Model validation 18](#_Toc153969130)

[Supplementary Appendix 9 – Additional results 20](#_Toc153969131)

[Supplementary Appendix 10 – Subgroup analysis 21](#_Toc153969132)

[Supplementary Appendix 11 – Best- and worst-case scenarios 22](#_Toc153969133)

[References: 23](#_Toc153969134)

# Supplementary Appendix 1 – information on SECA studies

The eligibility criteria from SECA ll were more restrictive and designed following the results from SECA l where they identified several negative predictive factors associated with poorer survival outcomes in patients. Compared to patients included in SECA l, patients selected for SECA II had smaller tumor diameter, nonprogressive disease on chemotherapy at time of liver transplantation, longer time from surgery of primary tumor to metastases, and lower carcinoembryonic antigen (CEA) levels prior to transplantation.^1^

# Supplementary Appendix 2 - Liver blood type distribution

During the model validation process, we identified that the waiting time for patients with blood type 0 was higher, while blood types A, B and AB were somewhat lower than the median waiting time from the empirical data. Therefore, we tweaked the blood type distribution that was assigned to the livers to obtain a median waiting time by blood type that similar to the empirical data. Our approach was to iteratively modify the initial distribution settings from the original distributions of 39% Type O, 49% Type A, 8% Type B, and 4% Type AB, to align with the Norwegian blood-type specific waiting times. We systematically tested various distributions, adjusting percentages specifically, such as to 39.5 % (original 39%) Type 0, 48.5 % (Original 0,49 %) type A, among others. This process was repeated multiple times, running simulations with each new distribution, until we aligned the median waiting time in our model with the blood type specific waiting times observed in Norway. The iterative approach allowed us to ensure that our simulation closely mirrored real-world waiting times, despite the lack of direct blood type distribution data.

As the blood type distribution between livers and patient will most likely not be identical in any yearly case, we do not think this harms the model. The final distribution of blood types for the liver transplants was: 44% Type O, 46.5% Type A, 7.5% Type B, and 2% Type AB.

# Supplementary Appendix 3– Quantile functions

To establish when certain events occurred in the mode, we used time-to event approach. Time of death and dropout on the waiting list, post-transplant survival, background mortality and survival when CRLM patients received palliative chemotherapy were estimated in the model using the quantile function for the most suitable parametric specification. **Table S1** lists the quantile function formulas used for deriving these event times when constructing the model.

| **Table S1:** Quantile function formulas used for deriving event times | |
| --- | --- |
| **Distribution** | **Formula** |
| ** Weibull* | ${[-ln(random number)/\lambda]}^{1/\gamma}$ |
| *** Log Logistic* | $\left\{ \ln\left[ -\gamma\ln\left( random number \right)+1 \right]-\lambda\right\}/\gamma$ |
| *Gompertz* | $\left\{ \ln\left[ -\gamma\ln\left( random number \right) \right]-\lambda\right\}/\gamma$ |
| * Scale and shape are exp(_cons) from the Stata output | |
| ** Scale is exp(-_cons) from the Stata output | |

# Supplementary Appendix 4 - Model assumptions

*Homogeneity in patient characteristics*

Due to limited data on outcomes by different patient characteristics, the population within the liver disease diagnosis were assumed to be homogeneous, except by blood type. Patients diagnosed with PBC, AC, Others and CRLM were assumed to have an age of 57 years, PSC and ALF were assumed to have an age of 43 years, and HCC patients were assumed to have an age of 61 years.^2^

*Waiting list death and dropout*

The patients that dropped out from the waiting list, regardless of reason for dropout, were assumed to die immediately upon removal from the model. This assumption was made due to data limitation and based on experts' opinions (SV and PDL). However, in reality, some patients drop out because of improved conditions—that occurs very seldom. We expect this assumption to no greatly impact that incremental impact between Strategy 1 and Strategy 2, as these individuals will become healthy independent of whether CRLM patients are enlisted.

Some assumptions were also made for specific liver disease diagnosis categories, which were stratified by the five most frequent liver disease diagnoses in Norway: Hepatocellular Carcinoma (HCC) (13.7%), Primary Biliary Cirrhosis (PBC) (6.7%), Primary Sclerosing Cholangitis (PSC) (18%), Acute Liver Failure (ALF) (8.3%) and Alcoholic Cirrhosis (AC) (10.1%). The remaining patients were pooled together in “Others”. **Table S2** shows the prevalence over diagnosis under the category “others”.

***Table S2:*** Prevalence of liver disease diagnosis under category “Other”

| Other |
| --- |
| Autoimmune cirrhosis |
| Metabolic diseases |
| Post-hepatitis B cirrhosis |
| Post-hepatitis C cirrhosis |
| Cryptogenic cirrhosis |
| Cirrhosis other cause |
| Polycystic liver disease |
| Cholangiocarcinoma |
| Secondary liver tumors |
| Other liver malignancies |
| Budd-Chiari |
| Biliary atresia |
| Cholestatic disease |
| Other liver diseases |

The HCC patients were assumed to have no time of dropout (only risk of death) while on the waiting list. This is because HCC patients usually receive medical treatment postponing the cancer spreading while waiting for a liver transplantation and will therefore be able to stay longer on the waiting list without experiencing death or dropout while waiting.^3^Further, the ALF patients were assumed not to have a time of death and dropout while on the waiting list since they are prioritized. In addition, individuals in the group “Other” were assigned the time of death and dropout based on all patients listed for a liver transplantation. Lastly, if the CRLM patients were eligible for the waiting list, then the time of death and dropout were the same as the survival function when they received palliative chemotherapy. In addition to withdrawals due to risk of death and dropout, the model assumed that 6% of the Status Quo patients were withdrawn from the waiting list. There are two reasons for this assumption: firstly, the data used for the risk of death or dropout was not sufficient to capture all the risk of death and dropout due to data availability. Secondly, some patients are withdrawn due to improved conditions. However, many patients have a risk of death or dropouts unconditionally upon waiting time i.e., enlisting CRLM patients will only affect the risk of death and dropout for some of the Status Quo patients. Therefore, we believe that the “harm” due to increased waiting time is sufficiently captured in the model.

*Matching and priority system*

The model assumed that matching between liver and patient was only dependent on blood type, and generally followed the queuing system “first in, first out”. However, following current practice, patients with ALF and patients listed for a re-transplant had a priority in the waiting list, and were therefore, assigned a liver as soon as a match was found.

*Re-transplantations*

The risk of re-transplantation is not differentiated among the diagnostic group. Also, listing for re-transplantation happened directly after the first transplantation. In addition, the patients who underwent a re-transplantation were assumed to have the same survival as when undergoing the first transplantation, which was due to a lack of data on the probability of re-transplantation and re-transplantation survival for CRLM patients. Subsequently, we assumed a small proportion of re-transplantations as a percentage and assumed that the patients who undergo a re-transplantation receive the same survival distribution as when undergoing the first transplantation. This assumption was made for both Status Quo and CRLM patients. In addition, we assumed that patients not eligible for a re-transplantation faced a survival time of 90 days, based on expert opinion.

*Post-transplant survival*

Post-transplant survival for all patients was assumed to be independent of the time on the waiting list^4^. In addition, the liver disease diagnosis category “Others” were assumed to have the general post-transplant survival as those undergoing liver transplantation in Norway.

The model also assumed that patients who survived 10 years post-transplant were subject to the Norwegian age-specific background mortality. This assumption was made due to available data on Kaplan Meier curves—where we observed a flattening of the curve after ten years ^2,5^; the Kaplan Meier curves showed that the risk of dying was greatest in the first 5 years post-transplant, after which the risk gradually decreases. Since the published Kaplan Meier curves were based on a population of age 0.1-74.1 years, while our model assumed a median age, and background mortality of the average age of individuals in our model (i.e., 67 years for PBC, AC and Others (57 + 10 years) or 53 years for PSC and ALF (43 + 10 years) was assigned after 10 years post-transplant. In this way, we believe that we were able to capture the risk of death post-transplant appropriately in the 10-year period post-transplant, before they were assigned a background mortality.

*Assumptions about Nordic VII and SECA II*

We assumed that the patients in SECA II, when not receiving a transplantation, had the same overall survival as the selected patients in the Nordic VII trial^.6^ This was recommended by experts in the field of oncology.

# Supplementary Appendix 5 - Reconstructing time-to-event data

We estimated survival times by extracting time of survival from published Kaplan Meier curves. The x- and y-values (in a survival curve plot) were extracted from the published curves using WebPlotDigitizer (see **Table S3** for the published curves used to construct time-to-event data). The x-value represented the follow-up time from the start of treatment, while y-values represented the survival probability at that certain time; these values allowed us to reconstruct individual time-to-event data from the Kaplan Meier curves. A cohort of 1000 patients was created for each Kaplan Meier curve to represent the start of the follow-up time, and based on the survival probability, the number of deaths in each time period was estimated. All the patients in the cohort were given an identification number with an estimated time-to-event.

| **Table S3:** Data sources used for survival analysis |  |  |
| --- | --- | --- |
| **Variables** | **Figure in source** | **Source** |
| **Status Quo patients** |  |  |
| Survival post-transplant |  |  |
| HCC | 14 | ^2^ |
| PBC | 2B | ^5^ |
| PSC | 14 | ^2^ |
| ALF | 2B | ^5^ |
| AC | 14 | ^2^ |
| Others | 2D | ^5^ |
|  |  |  |
| **CRLM patients** |  |  |
| Survival when receiving chemotherapy (NORDIC VII) | 2A | ^6^ |
| Survival post-transplant (SECA I) | 2A | ^6^ |
| Survival post-transplant (SECA II) | 1 | ^1^ |
|  |  |  |
| **Status Quo patients** |  |  |
| Death on waiting list |  |  |
| HCC | 1 | ^7^ |
| PBC | 2 | ^8^ |
| PSC | 2 | ^8^ |
| AC** | 2 | ^9^ |
| Others | 4 | ^10^ |
|  |  |  |
| Dropout on waiting list |  |  |
| PBC | 2 | ^8^ |
| PSC | 2 | ^8^ |
| AC** | 2 | ^9^ |
| Others | 4 | ^10^ |
|  |  |  |
| **CRLM patients** |  |  |
| Death and dropout on waiting list | 2A | ^6^ |
|  |  |  |
| **Background Mortality** |  |  |
| General for all | N/A | ^11^ |
| ** Figure included both death and deterioration | |  |

# Supplementary Appendix 6 - Survival analyses

Following NICE guidelines, we assessed the suitability for the different parametric specifications for all the 20 survival analyses. **Table S4** shows results from AIC & BIC, in addition **Table S5** shows an overview over the chosen parametric specification used to estimate survival and risk of drop out and death in our model. Survival data related to SECA l and ll studies needed to be extrapolated due to short follow-up period, **Figure S1a-*b*** shows the visual representation of the Kaplan Meier curve compared to the parametric distributions assessed. **Figure S2** shows the survival outcomes for the CRLM patients, and the Status Quo patents, used in the model.

| **Table S4:** Results from the AIC and BIC tests from the survival analyses | | | | |  |  |
| --- | --- | --- | --- | --- | --- | --- |
| **Post-Transplant Survival** | | | | | | |
| Statistical Test | Diagnosis | Status Quo Patients | | | | |
|  |  | *Exponential* | *Weibull* | *Log Normal* | *Log Logistic* | *Gompertz* |
| AIC | HCC | 1075 | 1065 | 1129 | 1108 | 1077 |
| BIC |  | 1079 | 1072 | 1137 | 1115 | 1084 |
| AIC | PBC | 958 | 946 | 980 | 965 | 960 |
| BIC |  | 962 | 953 | 987 | 973 | 968 |
| AIC | PSC | 1556 | 1555 | 1684 | 1608 | 1545 |
| BIC |  | 1560 | 1564 | 1692 | 1617 | 1554 |
| AIC | ALF | 1196 | 1036 | 1026 | 1035 | 1123 |
| BIC |  | 1200 | 1044 | 1033 | 1042 | 1131 |
| AIC | AC | 1548 | 1543 | 1777 | 1656 | 1484 |
| BIC |  | 1553 | 1551 | 1786 | 1665 | 1493 |
| AIC | Others | 975 | 965 | 963 | 964 | 965 |
| BIC |  | 979 | 973 | 971 | 972 | 974 |
| Statistical Test | Trial | CRLM Patients | | | | |
|  |  | *Exponential* | *Weibull* | *Log Normal* | *Log Logistic* | *Gompertz* |
| AIC | SECA I | 1052 | 1025 | 1037 | 1023 | 1035 |
| BIC |  | 1055 | 1035 | 1045 | 1032 | 1043 |
| AIC | SECA II | 593 | 590 | 571 | 585 | 595 |
| BIC |  | 597 | 598 | 579 | 595 | 601 |
| **Death on waiting list** | | | | | | |
| Statistical Test | Diagnosis | Status Quo Patients | | | | |
|  |  | *Exponential* | *Weibull* | *Log Normal* | *Log Logistic* | *Gompertz* |
| AIC | HCC | 272 | 267 | 268 | 257 | 268 |
| BIC |  | 276 | 276 | 276 | 276 | 277 |
| AIC | PBC | 337 | 335 | 333 | 335 | 330 |
| BIC |  | 341 | 344 | 342 | 344 | 339 |
| AIC | PSC | 490 | 491 | 488 | 491 | 488 |
| BIC |  | 495 | 502 | 499 | 502 | 499 |
| AIC | Others | 455 | 421 | 416 | 420 | 392 |
| BIC |  | 459 | 429 | 424 | 429 | 401 |
| **Dropout on waiting list** | | | | | | |
| Statistical Test | Diagnosis | Status Quo Patients | | | | |
|  |  | *Exponential* | *Weibull* | *Log Normal* | *Log Logistic* | *Gompertz* |
| AIC | PBC | 367 | 366 | 364 | 366 | 363 |
| BIC |  | 372 | 377 | 375 | 377 | 374 |
| AIC | PSC | 280 | 282 | 281 | 282 | 281 |
| BIC |  | 286 | 293 | 292 | 293 | 292 |
| AIC | Others | 372 | 356 | 354 | 356 | 353 |
| BIC |  | 376 | 365 | 362 | 364 | 361 |
| **Death + Dropout on waiting list** | | | | | | |
| Statistical Test | Diagnosis | Status Quo Patients | | | | |
|  |  | *Exponential* | *Weibull* | *Log Normal* | *Log Logistic* | *Gompertz* |
| AIC | AC | 1197 | 1103 | 1092 | 1100 | 1119 |
| BIC |  | 1201 | 1111 | 1101 | 1108 | 1127 |
| **Palliative chemotherapy** | | | | | | |
| Statistical Test |  | CRLM patients | | | | |
|  |  | *Exponential* | *Weibull* | *Log Normal* | *Log Logistic* | *Gompertz* |
| AIC |  | 1210 | 1106 | 1117 | 1055 | 1172 |
| BIC |  | 1214 | 1115 | 1125 | 1064 | 1180 |

| **Table S5:** The chosen parametric specifications for survival, background mortality, risk of death and risk of dropout on the waiting list. | | | | |
| --- | --- | --- | --- | --- |
| **Patients** | **Patient group** | **Survival** | **Risk of death** | **Risk of dropout** |
| Status Quo | HCC | Weibull | Weibull | Assumption |
|  | PBC | Weibull | Weibull | Weibull |
|  | PSC | Gompertz | Weibull | Weibull |
|  | ALF | Weibull | Assumption | Assumption |
|  | AC | Gompertz | Weibull | * |
|  | Other | Weibull | Weibull | Weibull |
| CRLM | SECA l | Log logistic | Log logistic | * |
|  | SECA ll | Log logistic | Log logistic | * |
|  | Palliative chemotherapy | Log logistic | N/A | N/A |
| * Risk of death and dropout is incorporated into the same curve using the specification in “Risk of death" column | | | | |
| N/A Not applicable | | | | |
|  |  |  |  |  |

**Figure S1:** Kaplan Meier curves related to CRLM patients survival. The KM curves are compared to different parametric specifications. Panel A, Extrapolation of survival data from the SECA l trial. Panel B, Extrapolation of survival data from the SECA ll trial.

**
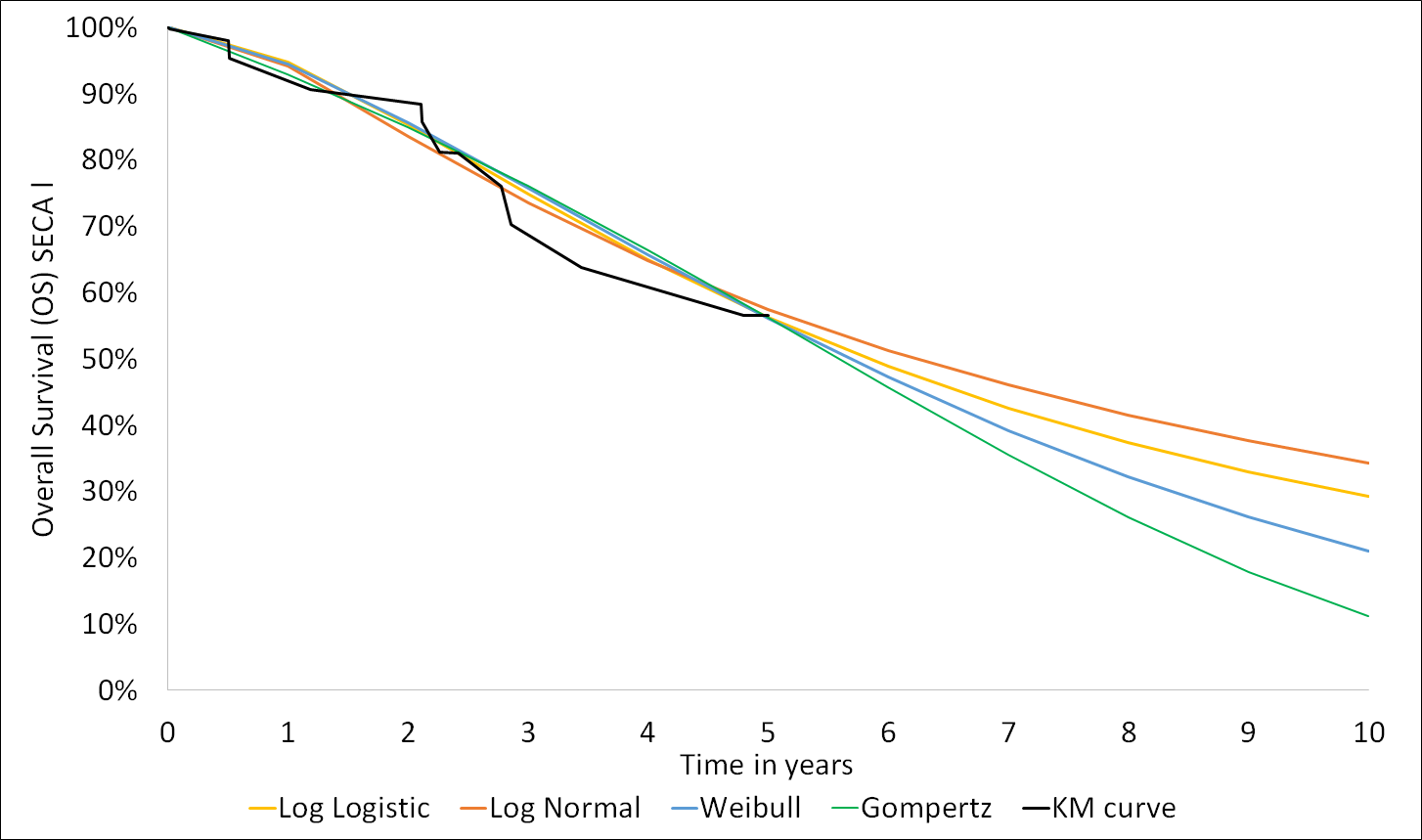
**

**A**

**
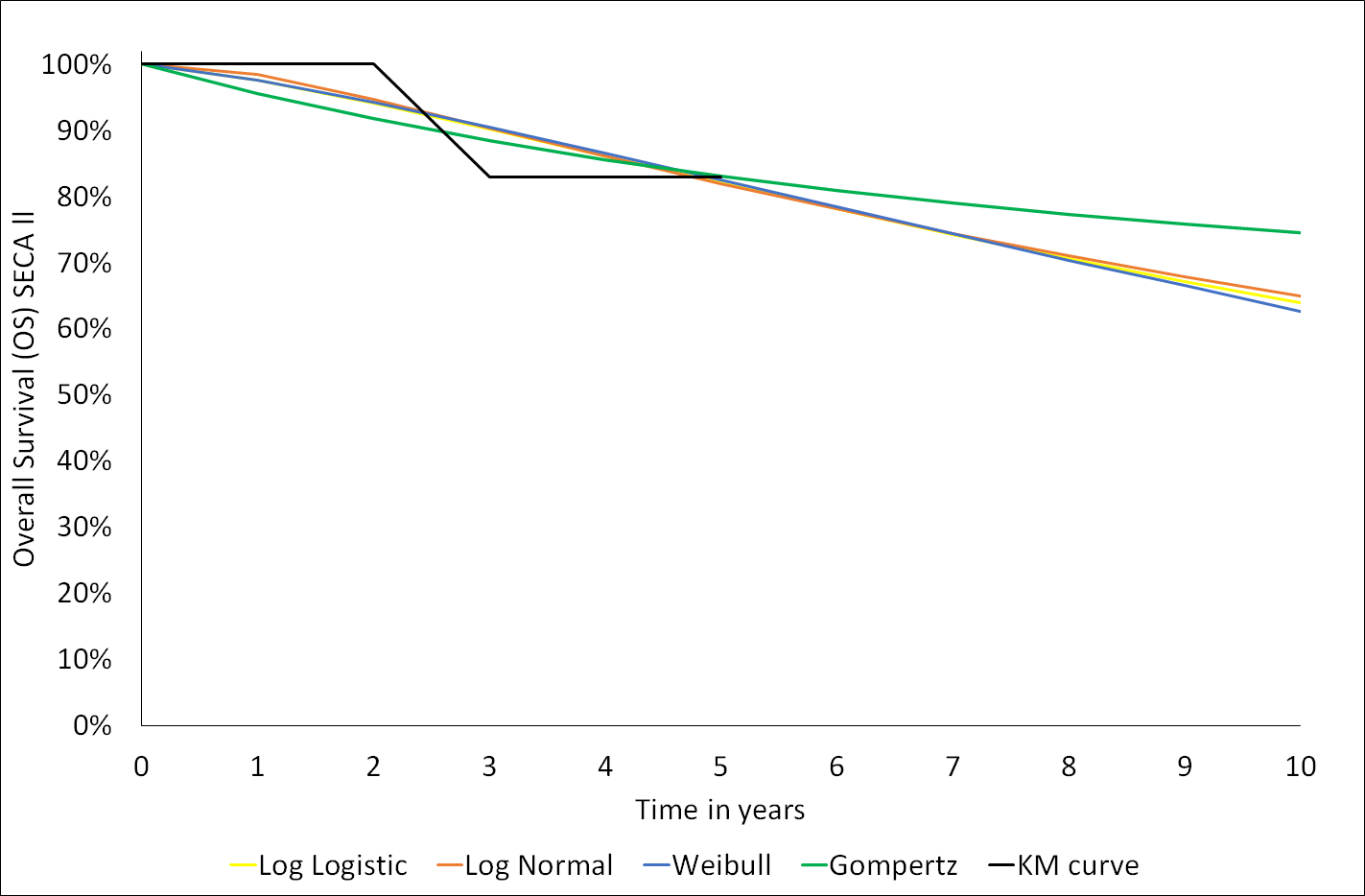
**

**B**

**Figure S2:** The curves represent the survival curves for the CRLM patients and the Status Quo patients used in the model.
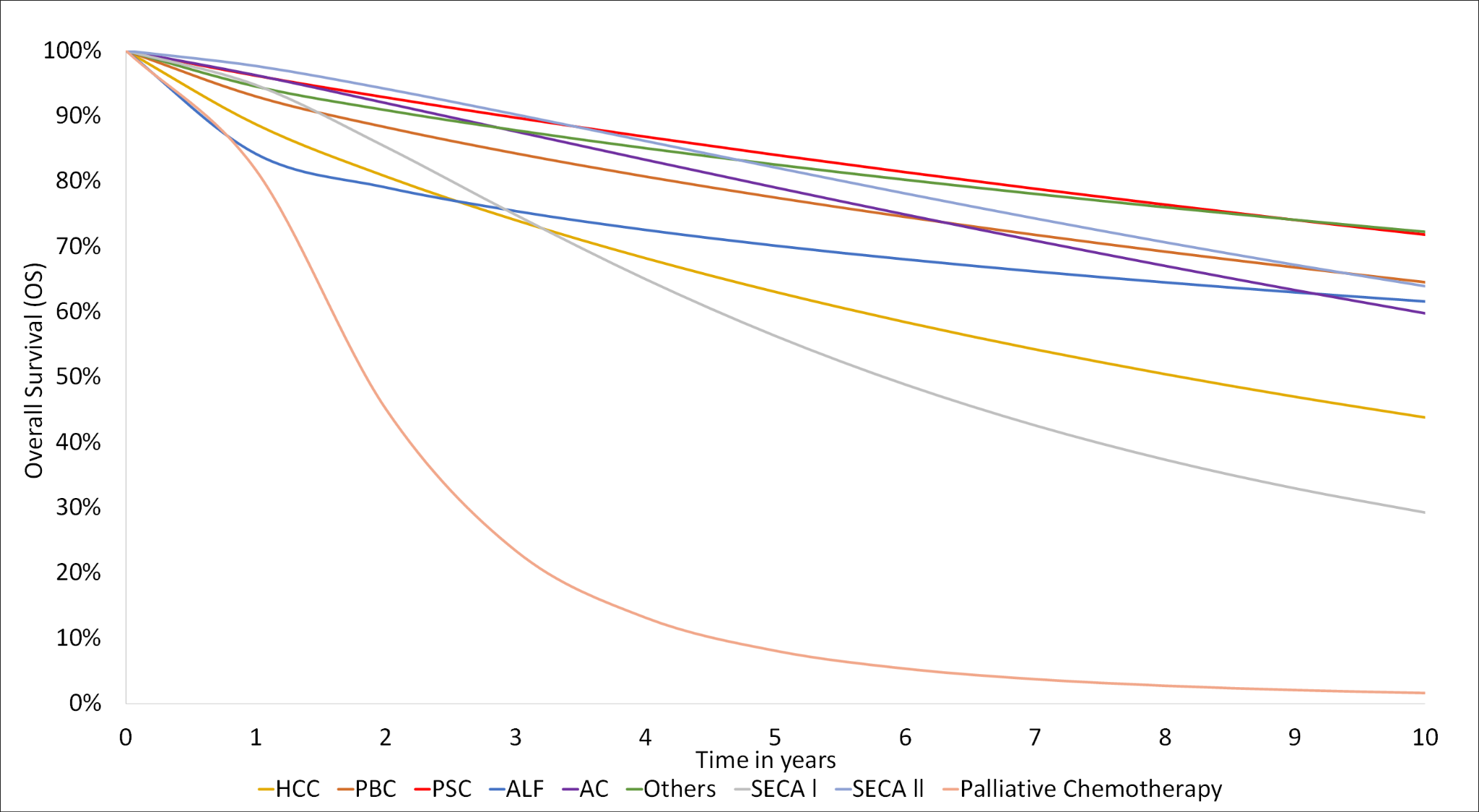


# Supplementary Appendix 7 - Stochastic uncertainty

*Determining optimal burn-in period*

To determine the optimal burn-in period we utilized Arena simulation software to analyze the stochastic uncertainty by making a new variable in Arena, Work in progress (WIP), that aggregated the total number of entities (livers, CRLM patients and Status Quo patients). Our objective was to ascertain the burn-in length to stabilize the transplantation queue prior to evaluating the impact of strategies. **Figure S3** shows the average number of entities at specific times across these replications. To balance computational burden and burn-in period, we noted that the replication data started to stabilize after 1900 days. After using a 1900-day burn-in period, the total average WIP was 19.5858 with a confidence interval half width of less than 0.14. This indicates a high level of precision in the simulations result.

**Figure S3:** Average WIP across 7000 replications over 7300 days (20 years).

Uncertainty analysis of burn-in period

To validate our chosen burn-in period, we conducted an uncertainty analysis. This involved experimenting with various warm-up lengths, specifically 5000, 10 000, and 30 000 days, and observing their impact on the median waiting time for patients. As documented in **Table S6**, we noticed a slight increase in waiting time, from 51.73 days with a 1900-day warm-up to 51.93 days with a 50,000-day warm-up. In addition, in **Figure S4**, we explored the effect of different warm-up lengths on the aggregated life years of introducing 5, 6, 7 and 8 CRLM patients, with the peak consistently occurring at 7 CRLM patients, irrespective of the warm-up duration.

Based on these findings, we observed only a slight change in median waiting time and aggregated life years. This outcome supported our choice of a 1900-day warm-up period.

**Table S6:** Median waiting time of base-case model (Strategy 1) using different warm-up length.

| **Warm up length (in days)** | **Median waiting time** |
| --- | --- |
| 1900 | 51.73 |
| 5000 | 51.75 |
| 7000 | 51.75 |
| 10 000 | 52.47 |
| 30 000 | 52.43 |
| 50 000 | 51.93 |

**Figure S4:** Net gain of introducing 5 to 8 CRLM patients using different warm-up length


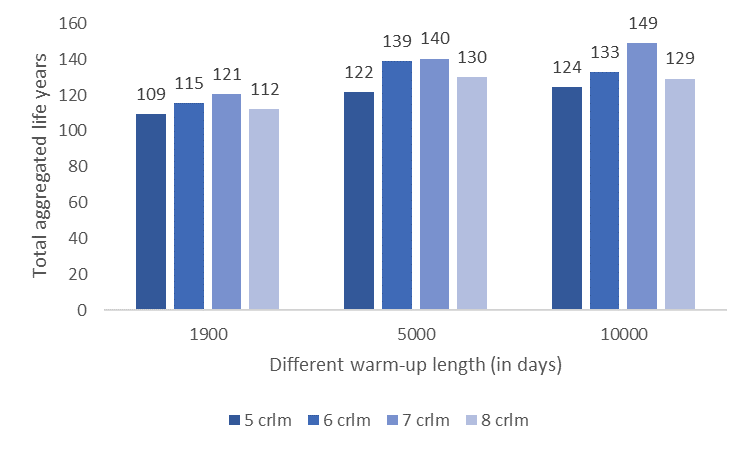


Choice of replication length

To determine the optimal number of replications, we systematically simulated the model using a range from 100 to 10,000 iterations. We considered the variability in life years for both Status Quo patients and CRLM patients, using Strategy 1 with 2 CRLM patients included in each iteration. Our analyses indicated that the variability in aggregated life years started to stabilize between 6000 and 8000 replications. To balance computational time and accuracy, we decided to use 7000 replications for all our analyses, as depicted in **Figure S5.**

**Figure S5:** The figure shows a control chart used to select the number of replications. The y-axis shows the aggregated life years for Status Quo patients and 2 CRLM patients in Strategy 1 and the x-axis shows the number of replications. The blue line represents the variation in life years in Strategy 1 for Status Quo patients and 2 CRLM patients, depending on the number of replications.


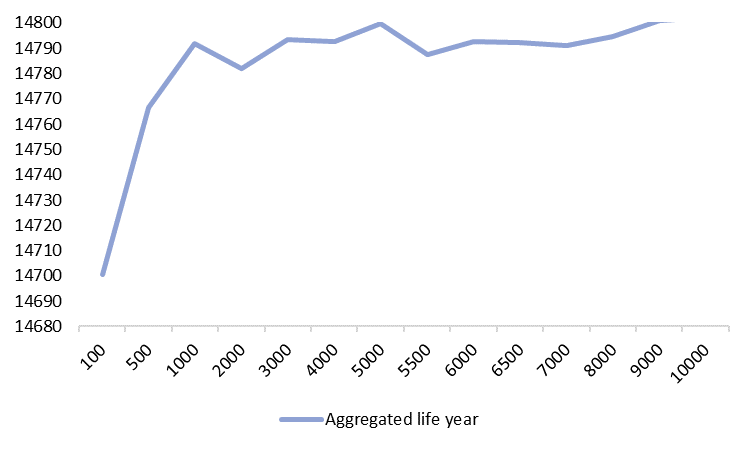


# Supplementary Appendix 8 - Model validation

*Face validity*

The model and analyses were presented for the Head of the Department of Transplantation Medicine at Oslo University Hospital (co-author PDL). Consensus was reached about the model's structure, assumptions, and inputs.

*Internal validity*

For internal validity, we examined the mathematical calculations used in our model:

a) We ensured that the equations we used for assigning time-to-event in our model was correct by ensuring that the predicted time given the random number in the quantile function matched the predicted time to event that we extracted from published Kaplan Meier curves when using the quantile function to assign patients randomly;

b) We continuously ensured that all model output matched expectations;

c) We inspected the average survival time for each patient group and the 90% credible interval and the patients related to each patient group with the minimum and maximum post-transplant survival time (**Table S7**);

d) We ensured that the minimum and maximum median waiting time for each patient in our base-case model was reasonable and in line with what we expected (**Table S8**).

| **Table S7**: Validation of survival estimates judging the survival time relatively to median age | | | | | |
| --- | --- | --- | --- | --- | --- |
|  | **Post-transplant survival time in years** | | | | |
| **Patient group (Median age)** | **Average** | **90 % credible interval** | **Min** | **Max** | **Max age** |
| HCC (62 yrs) | 8.7 | (0.2 - 24.1) | 0.0000 | 37.4 | 99.4 |
| PBC (57 yrs) | 13.1 | (0.2 - 30) | 0.0000 | 43.0 | 100.0 |
| PSC (43 yrs) | 25.2 | (0.5 - 45.4) | 0.0000 | 59.3 | 102.3 |
| ALF (43 yrs) | 20.1 | (0.1 - 44.6) | 0.0000 | 60.3 | 103.3 |
| AC (57 yrs) | 13.8 | (0.1 - 30.1) | 0.0000 | 43.3 | 100.3 |
| Others (57 yrs) | 14.2 | (0.2 - 30.3) | 0.0000 | 44.6 | 101.6 |
| Palliative chemotherapy (57 yrs) | 2.5 | (0.5 - 6.2) | 0.0155 | 12.2 | 69.2 |
| SECA l (57 yrs) | 8.3 | (0.42 - 26.9) | 0.0025 | 43.0 | 100.0 |
| SECA ll (57 yrs) | 13.4 | (0.5 – 42.7) | 0.0000 | 43.4 | 100.4 |

| **Table S8:** Internal validity of the waiting time variation within the blood groups | | |
| --- | --- | --- |
| **Median waiting time by bloodtype in days** | **Average** | **90% credible interval** |
| 0 | 120 | (6-284) |
| A | 43 | (2-124) |
| B | 68 | (3-187) |
| AB | 30 | (1-93) |
| Overall | 74 | (2 - 225) |

(e) Dependent Comparisons. We ensured that the equations we used for assigning time-to-event in our model was correct by ensuring that the predicted time given the random number in the quantile function matched the predicted time-to-event from the published Kaplan Meier curves. We also ensured that the model's survival output matched the survival from the extracted published Kaplan Meier curves by comparing and documenting in which time (reported in years) a 75 % survival rate was reached in the model compared to the published Kaplan Meier curves (**Table S9**).

(f) Dependent comparisons. We investigated and compared the median waiting type by blood type and the overall median waiting time in our model with Norwegian waiting time statistics. In addition, we compared the maximum and minimum waiting time in our model with the highest waiting time reported in Norway (**Table S10**).

| **Table S9:** Internal validation of survival estimates by patient group compared to empirical data | | | | |
| --- | --- | --- | --- | --- |
|  | **Time in years in where the different patient groups reached 75 % survival** | | | |
| **Patient group** | **Estimated KM curves used in model** | **Published KM curves (Empirical evidence)** | | **Difference (years)** |
| HCC | 2.20 | 2.32 | | -0.12 |
| PBC | 7.25 | 8.75 | | -1.5 |
| PSC | 8.94 | 8.71 | | 0.23 |
| ALF | 2.23 | 2.42 | | -0.19 |
| AC | 5.43 | 6.48 | | -1.05 |
| Others | 5.05 | 5.50 | | -0.45 |
| Palliative care | 1.18 | 1.29 | | -0.11 |
| SECA l | 2.64 | 2.83 | | -0.19 |
| SECA ll | 5.06 | N/A | | N/A |
| **Total difference for Status Quo patients** | |  | | -3,08 |
| **Total difference for CRLM patients** | |  | | -0.30 |
| N/A : data not available due to limited follow-up period of 5 years | | |  | |

| **Table S10:** Internal validity exploring the highest waiting time in days reported from the model outputs by blood type compared to the highest waiting time reported in Norway. | | |
| --- | --- | --- |
| **Blood type** | **Model output** | **Empirical evidence**  (Min – Max) |
| 0 | 794 | 366-427 |
| A | 390 | 366-427 |
| B | 537 | 366-427 |
| AB | 356 | 366-427 |

# Supplementary Appendix 9 – Additional results

| **Table S11:** The table shows the difference in life years from Strategy 1 to Strategy 2 for both Status Quo- and CRLM patients. The column labeled "Diff" represents the incremental effect between Strategy 1 and Strategy 2. | | | | | | | |
| --- | --- | --- | --- | --- | --- | --- | --- |
|  |  |  |  |  |  |  |  |
|  | **Life years** | | | | | | |
| **# CRLM patients**  **listed** | **SECA I criteria** | | |  | **SECA II criteria** | | |
|  | **Status Quo patients** | **CRLM patients** | **Difference** |  | **Status Quo patients** | **CRLM patients** | **Difference** |
| 1 | -25,8 | 56.6 | 30.8 |  | -31.3 | 107.2 | 75.9 |
| 2 | -63.2 | 114 | 50.7 |  | -62.5 | 212.1 | 149.6 |
| 3 | -91.1 | 170.2 | 79.0 |  | -91.4 | 318.0 | 226.6 |
| 4 | -128.7 | 225.0 | 96.4 |  | -133.3 | 421.0 | 287.7 |
| 5 | -170.7 | 280.1 | 109.4 |  | -171.2 | 523.1 | 351.9 |
| 6 | -218.5 | 333.8 | 115.3 |  | -218.9 | 627.2 | 408.3 |
| 7 | -264.2 | 384.9 | 120.7 |  | -275.5 | 724.8 | 449.6 |
| 8 | -326.0 | 438.2 | 112.1 |  | -326.9 | 825.5 | 498.6 |
| 9 | -376.4 | 485.6 | 109.2 |  | -380.8 | 916.6 | 535.8 |
| 10 | -449.8 | 537.0 | 87.2 |  | -443.4 | 1010.7 | 567.3 |

# Supplementary Appendix 10 – Subgroup analysis

A subgroup analysis was conducted on selected patients within the SECA I trial. When the SECA I trial was completed, the clinicians evaluated factors that were associated with reduced overall survival. Four prognostic factors were found to have significant association: tumor diameter above 5.5 cm, CEA levels before liver transplantation above 80 μg/L, time from surgery of primary tumor to metastases less than 2 years, and progressive disease on chemotherapy at time of liver transplantation. The subgroup analysis was based on this; a publication by Hageness et al..^12^ Hageness et al. have constructed Kaplan Meier curves for three groups of patients within SECA I, those having 0-1 prognostic factors, those having 2 and 3 prognostic factors, and those having all 4 prognostic factors. A subgroup analysis on those having 2 and 3 prognostic factors was conducted as these patients have a 5-year overall survival better than the overall SECA I patients; yet, poorer survival than SECA II patients. Under this inclusion criteria, approximately 3 CRLM patients would annually be qualified for the liver transplantation waiting list. Results of the heterogeneity analyses showing the aggregated life expectancy is shown in **Figure S6**.

**Figure S6.** Figure shows the aggregated life expectancy after conducting the heterogeneity analyses using SECA 1 (red line), SECA ll (green line) and subgroup analyses of SECA l subgroup analyses (blue line)

# Supplementary Appendix 11 – Best- and worst-case scenarios

Results of the best- and worst-case scenarios showed that median waiting times increased when the annual liver availability decreased. For example, when we assumed the expected annual number of CRLM patients was 2, the median waiting time increased from 98 to 123 days in the worst-case scenario (blood type A) with 17 fewer livers available than in our base case analysis. In contrast, the median waiting increased from 21 days to 24 days in the best-case scenario with 7 additional livers available than in our base case (**Table S12**). The student version of Arena Software was used to conduct analyses, which face several limitations, including not allowing more than 150 entities in the model. As a result, the number of livers and patients in the model cannot exceed 150 entities at the same time. Therefore, the structural uncertainty analysis conducted on best case and worst-case liver availability had to be performed by running the model for 7 years. In addition, this limitation made it impossible to conduct analyses on the most extreme values of liver and patient arrivals, which made it difficult to conduct a probabilistic analysis to explore parameter uncertainty.

| **Table S12:** Liver availability impact on median waiting time | | | | | |
| --- | --- | --- | --- | --- | --- |
| **Time between liver arrivals**  **(days)** |  | **Median waiting time by blood type (days)** | | | |
|  | ***# CRLM patients*** | **0** | **A** | **B** | **AB** |
| **Best case** | | | | | |
| 3.0 | 0 | 72 | 21 | 38 | 14 |
|  | 2 | 81 | 24 | 42 | 16 |
|  | 4 | 93 | 28 | 47 | 18 |
|  | 6 | 108 | 33 | 54 | 21 |
|  | 8 | 124 | 39 | 61 | 24 |
|  | 10 | 143 | 47 | 71 | 29 |
| **Base case** | | | | | |
| 3.18 | 0 | 106 | 32 | 53 | 21 |
|  | 2 | 123 | 38 | 61 | 24 |
|  | 4 | 145 | 46 | 71 | 28 |
|  | 6 | 166 | 57 | 82 | 34 |
|  | 8 | 193 | 70 | 95 | 41 |
|  | 10 | 219 | 87 | 110 | 50 |
| **Worst case** | | | | | |
| 3.55 | 0 | 248 | 98 | 123 | 56 |
|  | 2 | 283 | 123 | 145 | 70 |
|  | 4 | 318 | 149 | 166 | 85 |
|  | 6 | 354 | 181 | 192 | 105 |
|  | 8 | 386 | 212 | 217 | 123 |
|  | 10 | - | - | - | - |
| N/A: Not available due to software limitations (See description above) Liver arrival base case = 3.18 (114 available livers yearly) Liver arrival best case = 3.00 (121 available livers yearly) Liver arrival worst case = 3.55 (104 available livers yearly) | | | | | |

#

# References:

1. Dueland S, Syversveen T, Solheim JM, et al. Survival Following Liver Transplantation for Patients With Nonresectable Liver-only Colorectal Metastases. *Ann Surg*. 2020;271(2):212-218. doi:10.1097/SLA.0000000000003404

2. Melum E. The Nordic Liver Transplant registry (NLTR) - Annual report 2019. Published online 2020. http://www.scandiatransplant.org/members/nltr/TheNordicLiverTransplantRegistryANNUALREPORT2019.pdf

3. Yao FY, Bass NM, Nikolai B, et al. A follow-up analysis of the pattern and predictors of dropout from the waiting list for liver transplantation in patients with hepatocellular carcinoma: implications for the current organ allocation policy. *Liver Transpl*. 2003;9(7):684-692. doi:10.1053/jlts.2003.50147

4. Roberts MS, Angus DC, Bryce CL, Valenta Z, Weissfeld L. Survival after liver transplantation in the United States: A disease-specific analysis of the UNOS database. *Liver Transplantation*. 2004;10(7):886-897. doi:10.1002/lt.20137

5. Fosby B, Melum E, Bjøro K, et al. Liver transplantation in the Nordic countries - An intention to treat and post-transplant analysis from The Nordic Liver Transplant Registry 1982-2013. *Scand J Gastroenterol*. 2015;50(6):797-808. doi:10.3109/00365521.2015.1036359

6. Dueland S, Guren TK, Hagness M, et al. Chemotherapy or Liver Transplantation for Nonresectable Liver Metastases From Colorectal Cancer? *Annals of Surgery*. 2015;261(5):956-960. doi:10.1097/SLA.0000000000000786

7. Salvalaggio PR, Felga G, Axelrod DA, Guardia BD, Almeida MD, Rezende MB. List and Liver Transplant Survival According to Waiting Time in Patients With Hepatocellular Carcinoma. *American Journal of Transplantation*. 2015;15(3):668-677. doi:10.1111/ajt.13011

8. Singal AK, Fang X, Kaif M, et al. Primary biliary cirrhosis has high wait-list mortality among patients listed for liver transplantation. *Transpl Int*. 2017;30(5):454-462. doi:10.1111/tri.12877

9. Thuluvath PJ, Hanish S, Savva Y. Waiting List Mortality and Transplant Rates for NASH Cirrhosis When Compared With Cryptogenic, Alcoholic, or AIH Cirrhosis. *Transplantation*. 2019;103(1):113-121. doi:10.1097/TP.0000000000002355

10. Kim WR, Therneau TM, Benson JT, et al. Deaths on the liver transplant waiting list: An analysis of competing risks. *Hepatology*. 2006;43(2):345-351. doi:https://doi.org/10.1002/hep.21025

11. 07902: Dødelighetstabeller, etter kjønn, alder x, statistikkvariabel og år. Statistikkbanken. Accessed March 24, 2021. https://www.ssb.no/statbank/table/07902/tableViewLayout1/

12. Hagness M, Foss A, Line PD, et al. Liver Transplantation for Nonresectable Liver Metastases From Colorectal Cancer. *Annals of Surgery*. 2013;257(5):800-806. doi:10.1097/SLA.0b013e3182823957
